# Long-term retinal oximetry and OCT angiography in patients recovered from COVID-19

**DOI:** 10.1038/s41598-025-21794-w

**Published:** 2025-10-29

**Authors:** Zuzana Schreiberová, Samuel Genzor, Tomáš Mudroch, Barbora Šindelářová, Petra Hübnerová, Miroslava Malušková, Klára Marešová, Martin Šín, Marta Karhanová

**Affiliations:** 1 Department of Ophthalmology, Faculty of Medicine and Dentistry, Palacky University Olomouc, Olomouc, Czech Republic; 2 Department of Ophthalmology, University Hospital Olomouc, Olomouc, Czech Republic; 3 Department of Respiratory Diseases and Tuberculosis, University Hospital Olomouc, Olomouc, Czech Republic; 4 Department of Respiratory Diseases and Tuberculosis, Faculty of Medicine and Dentistry, Palacky University Olomouc, Olomouc, Czech Republic; 5 Faculty of Medicine and Dentistry, Center for Digital Health, Palacky University Olomouc, Olomouc, Czech Republic; 6 Department of Ophthalmology, 1st Faculty of Medicine, Military University Hospital Prague, Charles University, Prague, Czech Republic

**Keywords:** Coronavirus disease 19, Retinal oximetry, Oxymap, Optical coherence angiography, Mechanical ventilation, Extracorporeal membrane oxygenation, Retinal diseases, Ocular ischemic syndrome

## Abstract

The aim of this study was to analyse retinal microvascular abnormalities in patients with various degrees of severity of the course of Coronavirus Disease 2019 (COVID-19), with a focus on patients requiring extracorporeal membrane oxygenation (ECMO) or mechanical ventilation (MV). We subclassified the patients after COVID-19 into 3 groups based on the severity of the disease and then performed standard ophthalmic examination, optical coherence tomography (OCT), macular OCT angiography (OCT-A) and retinal oximetry (RO). A total of 32 patients (21 men and 11 women; mean age of 51 years) were included in the study. Group 1 (mild COVID-19 course) included 11 patients, group 2 (moderate course) included 8 patients, and group 3 (severe course) included 13 patients which is particularly noteworthy. The median time after COVID-19 recovery was 22 months. No statistically significant difference between the groups was detected in any of the parameters of interest, suggesting resilience of retinal vasculature post-COVID-19. Patients after full recovery from severe COVID-19 do not show any significant decrease in oxygen saturation of retinal vessels 1.5 years (median) after the episode. Our data show that even a severe course of COVID-19 with ECMO or MV does not cause chronic microvascular changes in the retina.

## Introduction

Coronavirus disease 19 (COVID-19) is caused by a new coronavirus called Severe acute respiratory syndrome coronavirus 2 (SARS-CoV-2). The first pneumonia cases of this aetiology appeared in December 2019 in Wuhan, China^1^. The virus spread throughout the world and, in March 2020, the WHO officially declared the beginning of the COVID-19 pandemic^2^.

SARS-CoV-2 is able to invade various human tissues, with the primary entry point being the respiratory tract. It is associated with microvascular involvement and endothelial dysfunction (ED). According to various studies, these affections develop in virtually all infected patients, except in critically ill patients, although this distinction is not clinically significant^3–5^.

The endothelium is a single-layered lining of blood vessel inner walls, which plays an important role in haemocoagulation regulation. Its physiological function is to provide a non-wetting surface of the vessels, regulate blood clotting and control the vessel wall tension. There are also paracrine functions—it is responsible for the production of vasoconstrictive and vasodilating mediators and various cytokines. It also degrades several vasoactive substances. The main characteristics of ED are endothelial damage leading to increased vascular permeability and an imbalance between vasoactive effects, resulting in vasospastic and prothrombogenic mechanisms dominance^6^. ED arises as a result of various infectious and non-infectious diseases (diabetes mellitus, arterial hypertension, dyslipidaemia). In COVID-19 infection, ED is caused by a combination of several factors—direct damage caused by the virus, oxidative stress, impaired coagulation, and the response of the immune system to the presence of the virus^7^.

SARS-CoV-2 enters the cell using angiotensin-converting enzyme 2 (ACE-2) receptor and transmembrane serine proteinase 2 (TMPRSS-2). After the attachment of the viral particle to the host cell using the S1 domain of the spike protein, the spike protein is lysed through TMPRSS-2 into its two subunits. The S2 subunit subsequently induces membrane fusion and virion endocytosis^8–10^. Both key proteins exhibit substantial expression not only on the cells of the respiratory tract, but also in the heart, kidney, gastrointestinal tract, on the epithelium of the cornea, conjunctiva and endothelium of vessels, including retinal vessels^11,12^. This leads to the release of inflammatory cytokines and development of endotheliitis, which causes a wide range of signs stemming from the posterior segment. Individual case reports or case series have described cases of acute macular neuroretinopathy^13^ or paracentral acute middle maculopathy^14^, but the most numerous group of manifestations are microvascular abnormalities such as soft exudates, microhaemorrhages and increased tortuosity of blood vessels^15,16^.

Previous studies demonstrated the significance of OCT and OCT-A in the analysis of vessel density (VD) in the macular area, the size of the foveal avascular zone (FAZ) or the thickness of the retinal layers in post-COVID-19 individuals^17–23^. However, only one of the published studies so far focused on the eventual changes in the oxygen saturation of retinal vessels in these patients^24^. Retinal oximetry (RO) is a non-invasive imaging method that has become of substantial interest in recent years. It enables measurement of oxygen saturation of arteries, veins and arterio-venous difference of retinal vessels, which can tell us more about the metabolic activity in a given retinal area. It is also able to measure arterial and venous diameters, which can serve as potential biomarkers for cardiovascular disease^25^. In contrast, OCT-A shows in detail mainly structural microvascular changes at the capillary level.

Oxygen saturation measurement in the retinal vessels is based on the fact that the light absorbance of the vessel depends on the wavelength and is affected by the amount of oxyhaemoglobin and deoxyhaemoglobin. At a wavelength of 570 nm, arteries and veins exhibit almost identical appearance due to the similarity of the light absorbance of oxygenated and non-oxygenated haemoglobin. Conversely, at a wavelength of 600 nm (and many other wavelengths), there is a substantial difference in the absorbance of both oxyhaemoglobin and deoxyhaemoglobin (the absorbance of deoxyhaemoglobin is higher than the absorbance of oxyhaemoglobin). Therefore, arteries are significantly darker than veins. The intensity of selected points on the images acquired at these two wavelengths is measured and an oxygen saturation value is assigned to each point in the vessel^26–28^.

The aim of the presented study is to use RO, OCT and OCT-A to analyse and quantify possible retinal microvascular abnormalities in individuals after COVID-19, as this disease may lead to possible post-inflammatory damage to the vascular endothelium. Furthermore, we wanted to compare the group with mild, moderate and severe course of the disease with a focus to the critically ill group, which included mainly patients after extracorporeal membrane oxygenation (ECMO) or mechanical ventilation (MV). ECMO is a therapeutic modality utilised in cases of life-threatening cardiac or respiratory failure when other methods, including cardiopulmonary resuscitation, failed to achieve spontaneous circulation^29^. The first time it was used was in 1972 in a patient with post-traumatic respiratory failure^30^. Retinal symptoms in patients after ECMO were the focus of only a few of reports, mostly studies on paediatric patients^31–33^. Moreover, we wanted to evaluate an eventual association between the RO parameters of interest and OCT or OCT-A.

## Material and methods

Patients under regular follow-up at the centre for patients after COVID-19 at the Department for Respiratory Diseases and Tuberculosis of the University Hospital Olomouc and the Faculty of Medicine of the Palacký University in Olomouc were enrolled.

The following inclusion criteria were required: age over 18 years, a history of COVID-19, transparent ocular media allowing for fundus photography, signed informed consent to enter the study. Exclusion criteria were as follows: a previously known eye disease affecting retinal oxygen saturation (diabetic retinopathy, vascular occlusions, use of antiglaucoma agents), any other serious eye disease (advanced or uncontrolled glaucoma, retinitis pigmentosa, age-related macular degeneration, eye tumors), reduced transparency of the ocular media limiting fundus visualisation and preventing high-quality image acquisition (severe dry eye syndrome, corneal scars, significant cataract, significant vitreous opacities, haemophthalmos), significant refractive error preventing sharp image examinations (a refractive error greater than ± 6.0 spherical dioptres or ± 3.0 cylindric dioptres), history of intraocular surgery with the exception of cataract surgery more than 3 months before the enrolment, intravitreal administration of drugs against vascular endothelial growth factor or retinal laser photocoagulation, history of eye injury, acute eye infection, known systemic disease affecting retinal oxygen saturation or leading to alterations of ocular fundus (chronic obstructive pulmonary disease, multiple myeloma, malignant hypertension, hyperglobulinemia), pregnancy, breastfeeding.

All patients underwent visual acuity examination using Early Treatment Diabetic Retinopathy Study (ETDRS) or Snellen optotypes, intraocular pressure measurement with a Canon TX-20P non-contact tonometer (Canon Medical Systems Europe BV, Amstelveen, The Netherlands), biomicroscopic examination of the anterior segment of the eye and the examination of the posterior segment of the eye after the administration of short-term mydriatics (1% tropicamide and 10% phenylephrine) using a slit lamp, macular OCT and OCT-A using Spectralis Optical Coherence Tomography Plus version 1.11.2.0 (Heidelberg Engineering, Heidelberg, Germany) and RO using an Oxymap T1 automatic retinal oximeter (Oxymap ehf., Reykjavík, Iceland).

OCT scans were acquired using Heidelberg Spectralis Software version 7.0.1 and centred on the patient’s fovea. OCT scan patterns were as follows: Number of B-scans = 61, Pattern Size = 30**˚** × 25**˚**, Distance between B-scans = 124 µm, ART images average = 10 frames. We subtracted individual layer thicknesses from the Posterior Pole Analysis in the central subfield. We included central retinal thickness (CRT), inner retinal layer thickness (IRL), ganglion cell layer thickness (GCL), and subfoveolar retinal nerve fibre layer thickness (RNFL) in the statistics. IRL is defined by Heidelberg Spectralis Software as the difference between this two: (internal limiting membrane plus external limiting membrane) and (outer plexiform layer plus external limiting membrane). Furthermore, we took a radial RNFL scan centred at the optic nerve (ON) disc, circle diameter = 12° (3.6 mm), ART images average = 100 frames.

OCT-A was performed over a square section, size 20**˚** × 20**˚**, centred in the patient’s fovea. OCT scan patterns were as follows: Number of B-Scans: 512, Pattern Size = 20**˚** × 20**˚**, Distance between B-scans: 11 µm, ART images average = 9 frames. In addition, we excluded patients with a history of diabetes mellitus (DM) and patients with low scan quality or no scan in the Posterior Pole Analysis mode from OCT-A analysis compared to RO. After the inspection of segmentation, we focused on the VD in the capillary plexuses in the macula—retinal nerve fibre layer vascular plexus (NFLVP), superficial capillary plexus (SCP), intermediate capillary plexus (ICP), deep capillary plexus (DCP), choriocapillaris (CC), superficial vascular complex (SVC) and deep vascular complex (DVC). Furthermore, FAZ parameters were evaluated in the SVC and DVC—size (mm^2^), circumference (mm), circularity, roundness.

**Fig. 1:**
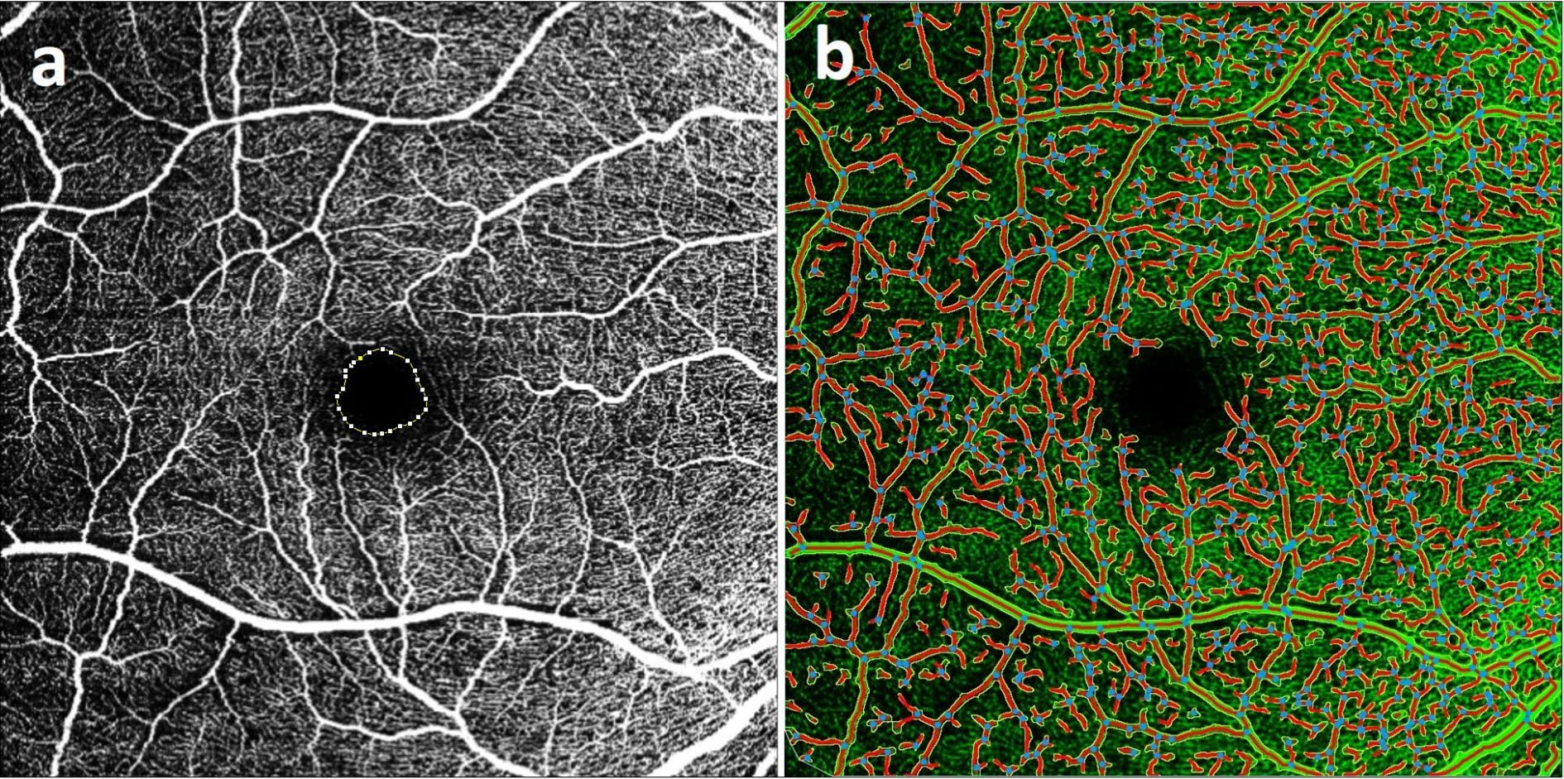
Superficial vascular plexus; (**a**) macular optical coherence angiography in ImageJ software, measurement of foveal avascular zone parameters: area 0.357 mm^2^, perimeter 2.286 mm, circularity 0.868, roundness 0.898 (Spectralis Optical Coherence Tomography Plus version 1.11.2.0, Heidelberg Engineering, Heidelberg, Germany); (**b**) vessel density analysis of the same patient in AngioTool.

VD was analysed using AngioTool version 0.6a (National Institutes of Health, National Cancer Institute, Bethesda, Maryland, USA; Fig. 1)^21,34^. Measurement parameters were set according to a previous study: threshold parameters = 30 and 255, vessel thickness = 5, removal of small particles = 80^21^. VD was defined as the ratio of the vessel area to the total measured area. VD was evaluated in the entire macular scan using an external software package, as no OCT device with analytical software was available for such evaluation. Therefore, macula was not divided into individual sectors according to ETDRS grid and VD was not calculated in individual segments contrary to other studies^18,19,22^, which might have led to more accurate results.

FAZ parameters were measured using ImageJ version 1.54d (National Institutes of Health, Bethesda, Maryland, USA; Fig. 1). The measurement scale was calibrated based on known image dimensions and FAZ boundaries were manually drawn using a free selection tool. We focused on FAZ area, circumference, circularity and roundness. Circularity refers to the degree of similarity of FAZ to a perfect circle. A value of 1.0 represents a perfect circle. The smaller this value, the less circular the shape is. Roundness indicates the most corresponding ellipse, and additionally considers irregularities around FAZ perimeter^35^. FAZ measurement and analysis were performed by two independent investigators in SVC and DVC layers. Their mean values were included in the analysis.

**Fig. 2:**
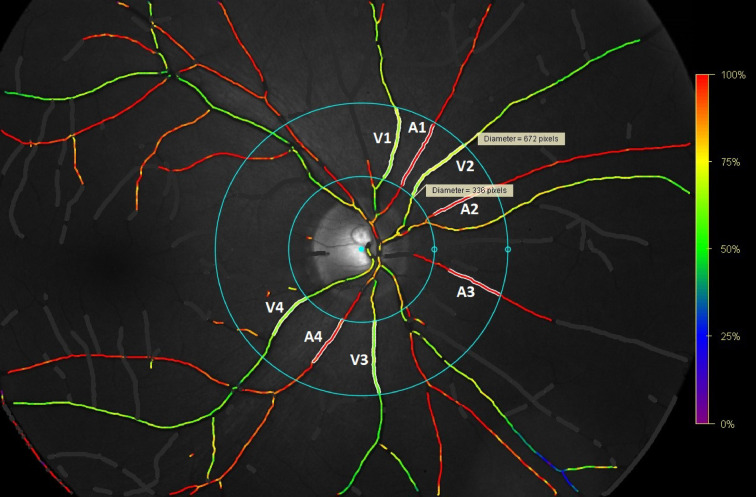
Retinal oximetry analysis, analysed vessels marked in white (Oxymap T1 automatic retinal oximeter, Oxymap ehf., Reykjavík, Iceland). A1–A4 = artery 1–4, V1–V4 = vein 1–4, diameters: V1 149 µm, V2 115 µm, V3 155 µm, V4 139 µm, A1 88 µm, A2 111 µm, A3 76 µm, A4 122 µm.

Automatic retinal oximeter is a device paired with Topcon TRC-50DX retinal camera (Topcon Corporation, Tokyo, Japan). This device acquires two retinal images of different wavelengths simultaneously. The intensity of selected points on the images is measured and oxygen saturation value is assigned to each point in the vessel (Fig. 2). The parameters of interest included arterial and venous saturation of retinal vessels, arterio-venous (AV) difference and arteriole and venule diameter. The measurement was performed under standardised conditions according to a specific protocol (Protocol for acquisition and analysis of Oxymap T1 oximetry images, version from 21/11/2013). Oxymap Analyzer Software, version 2.4.0 was used to calculate haemoglobin oxygen saturation. RO analysis was performed by one examiner in all the subjects. The analysis only included one patient eye at a time, primarily the right eye, to avoid potential bias. The left was only used if the quality of right eye scan was too low.

We divided the patients into 3 groups based on COVID-19 severity according to European Respiratory Society guidelines^36^—group 1 included patients with a mild COVID-19 course, without the need for hospitalisation; group 2 included patients who required hospitalisation and low-flow oxygen therapy, and group 3 patients requiring high-flow oxygen therapy, non-invasive or invasive ventilation or ECMO. The aim of the study was to compare the above-described OCT, OCT-A and RO parameters between groups and to evaluate the correlation between AV difference and OCT and OCT-A parameters in individual groups.

The study protocol was approved on 16/1/2023 by the Ethics Committee of the University Hospital Olomouc and the Faculty of Medicine of the Palacký University in Olomouc (Reference number 9/23). All examinations were performed in accordance with good clinical practice and the Declaration of Helsinki. All patients signed an informed consent with the participation in the study. The study was registered at Clinical Trials under number NCT05747547.

Statistical software IBM SPSS Statistics version 23 (Armonk, NY: IBM Corp.) was used for data analysis. The Kruskal–Wallis test and Mann–Whitney post-hoc test were used to compare quantitative parameters in groups delineated based on COVID-19 severity. The analysis of covariance (ANCOVA) method was used to adjust for potential confounders (e.g. age, hypertension). Bonferroni correction was used for multiple comparisons in post-hoc tests. Fisher’s exact test was used to compare qualitative parameters between the groups. Dependence between quantitative parameters was analysed using Spearman’s correlation coefficient. Data normality was assessed using the Shapiro–Wilk test. All tests were considered at the significance level of 0.05.

## Results

The cohort included 34 patients. One patient was excluded due to newly diagnosed non-proliferative diabetic retinopathy and one patient due to dry form of age-related macular degeneration. The final cohort for the analysis of RO parameters consisted of 32 patients, 21 (66%) men and 11 (34%) women with a mean age of 51.4 ± 13.3 (s.d.) years. The median time after COVID-19 recovery was 22 months (range of 1–27). The right eye was used for the analysis in 24 patients (75%); the left eye in 8 patients (25%). Group 1 (mild course of the disease) comprised 11 patients, group 2 (moderate course) 8 patients and group 3 (severe course) 13 patients. Mean visual acuity in logMAR scale was 0.03, mean intraocular pressure was 16.84 torr.

The final cohort for the analysis of OCT-A comprised a total of 22 patients, 13 (59%) men and 9 (41%) women with a mean age of 48.9 ± 12.8 (s.d.) years. The right eye was used for the analysis in 15 patients (68%); the left eye in 7 patients (32%). Group 1 (mild course of the disease) comprised 9 patients, group 2 (moderate course) 6 patients and group 3 (severe course) 7 patients.

**Fig. 3:**
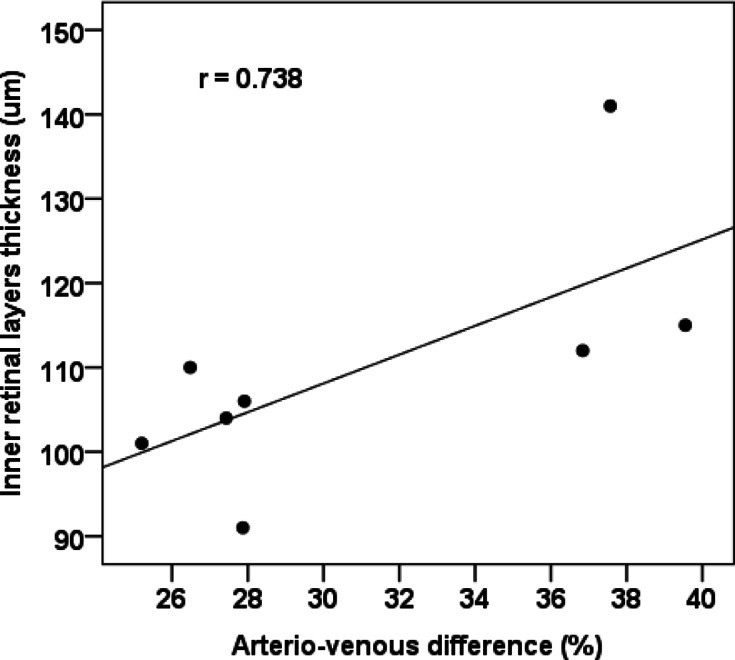
Correlation of the arterio-venous difference and the inner retinal layers thickness in patients with moderate disease course.

Basic demographic and clinical characteristics of the individual groups are presented in Table 1. There was no statistically significant difference between the groups when comparing potential confounders (e.g. age, hypertension). No statistically significant difference between the groups was found in any of the OCT, OCT-A or RO parameters of interest (Tables 2 and 3). A strong, statistically significant positive correlation was demonstrated between the AV difference and IRL thickness (r = 0.738; Fig. 3).

**Table 1:** Basic demographic and clinical characteristics of the groups according to the severity of the disease course.

	Course of the disease			*p* value*
	Mild	Moderate	Severe	
Age (years, median)	51.0	53.5	62.0	0.738
Sex (male)	72.7%	50.0%	69.2%	0.642
Eye (right)	81.8%	87.5%	61.5%	0.387
Cataract surgery (yes)	0.0%	0.0%	7.7%	1
Arterial hypertension (yes)	27.3%	25.0%	38.5%	0.795
Diabetes mellitus (yes)	9.1%	12.5%	15.4%	1
Dyslipidaemia (yes)	9.1%	50.0%	38.5%	0.131
Smoking (yes)	9.1%	0.0%	0.0%	0.594
Recovery time from COVID-19 (months, median)	20.0	23.0	22.0	0.316

*age and recovery time from COVID-19—Kruskal–Wallis test; other categories—Fisher’s exact test.

**Table 2:** Comparison of groups according to disease severity in retinal oximetry and optical coherence tomography parameters.

	Course of the disease			Kruskal–Wallis test *p* value
	Mild	Moderate	Severe	
SpO_2_ venous (%)	64.3	64.8	56.4	0.447
SpO_2_ arterial (%)	94.9	92.7	93.5	0.306
AV difference (%)	30.2	27.9	35.4	0.585
Diameter of arterioles (µm)	99	110	116	0.216
Diameter of venules (µm)	157	142	159	0.277
CRT fovea (µm)	289	290	276	0.632
IRL fovea (µm)	97	108	96	0.315
RNFL fovea (µm)	13.5	14.0	13.0	0.362
GCL fovea (µm)	15.5	19.0	16.0	0.249
RNFL disc (µm)	93	105	102	0.031*

Data in the table are presented as median. SpO_2_ = oxygen saturation, AV = arterio-venous, CRT = central retinal thickness, IRL = inner retinal layers, RNFL = retinal nerve fibre layer, GCL = ganglion cell layer. *post-hoc tests showed no significant difference.

**Table 3:** Comparison of groups according to disease severity in optical coherence angiography parameters.

	Course of the disease			Kruskal–Wallis test *p* value
	Mild	Moderate	Severe	
FAZ SVC area (mm^2^)	0.395	0.246	0.395	0.315
FAZ SVC perimeter (mm)	2.276	1.979	2.370	0.237
FAZ SVC circularity	0.877	0.899	0.887	0.744
FAZ SVC roundness	0.886	0.895	0.913	0.752
FAZ DVC area (mm^2^)	0.239	0.159	0.202	0.498
FAZ DVC perimeter (mm)	1.902	1.498	1.679	0.408
FAZ DVC circularity	0.888	0.896	0.887	0.394
FAZ DVC roundness	0.823	0.879	0.896	0.601
VD NFLVP	17.1	19.1	15.0	0.309
VD SCP	30.4	32.3	30.6	0.410
VD ICP	27.6	28.6	22.1	0.398
VD DCP	35.3	37.5	35.8	0.709
VD CC	41.6	36.4	39.6	0.469
VD SVC	25.7	28.0	25.4	0.411
VD DVC	25.8	27.9	19.8	0.885

Data in the table are presented as median. FAZ = foveal avascular zone, VD = vessel density, SVC = superficial vascular complex, DVC = deep vascular complex, NFLVP = retinal nerve fibre layer vascular plexus, SCP = superficial capillary plexus, ICP = intermediate capillary plexus, DCP = deep capillary plexus, CC = choriocapillaris.

**Fig. 4:**
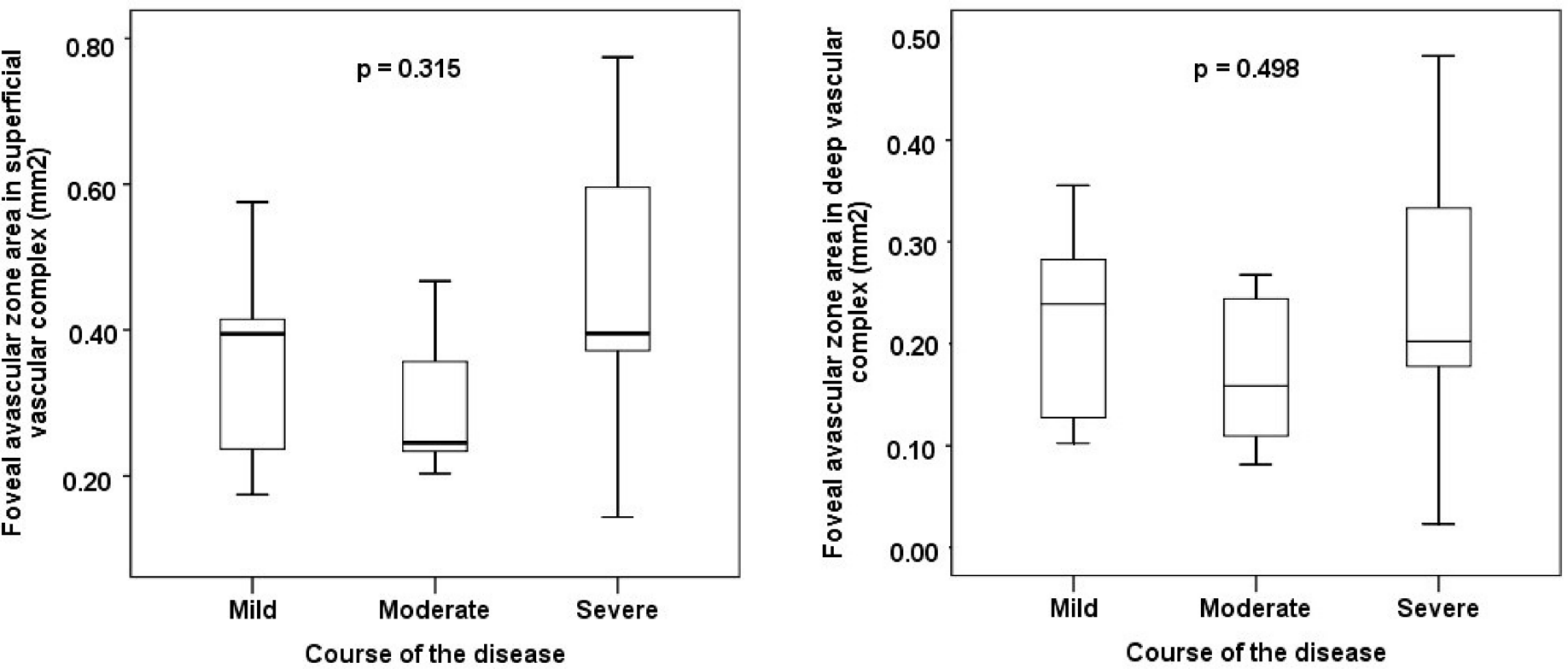
Comparison of foveal avascular zone area size in the superficial and deep vascular complex between groups.

**Fig. 5:**
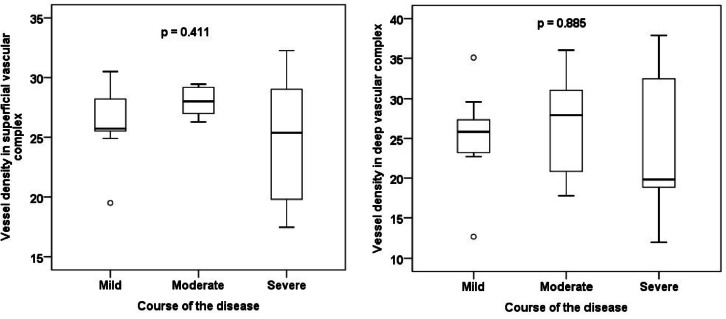
Comparison of vessel density in the superficial and deep vascular complex between groups.

**Fig. 6:**
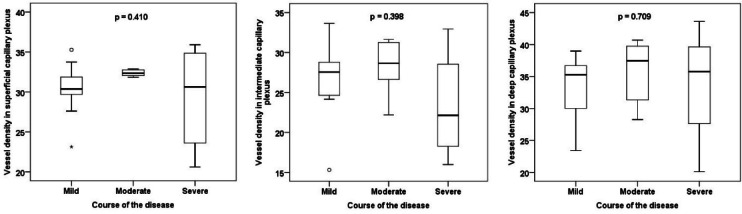
Comparison of vessel density in the superficial, intermediate and deep capillary plexus between groups.

**Fig. 7:**
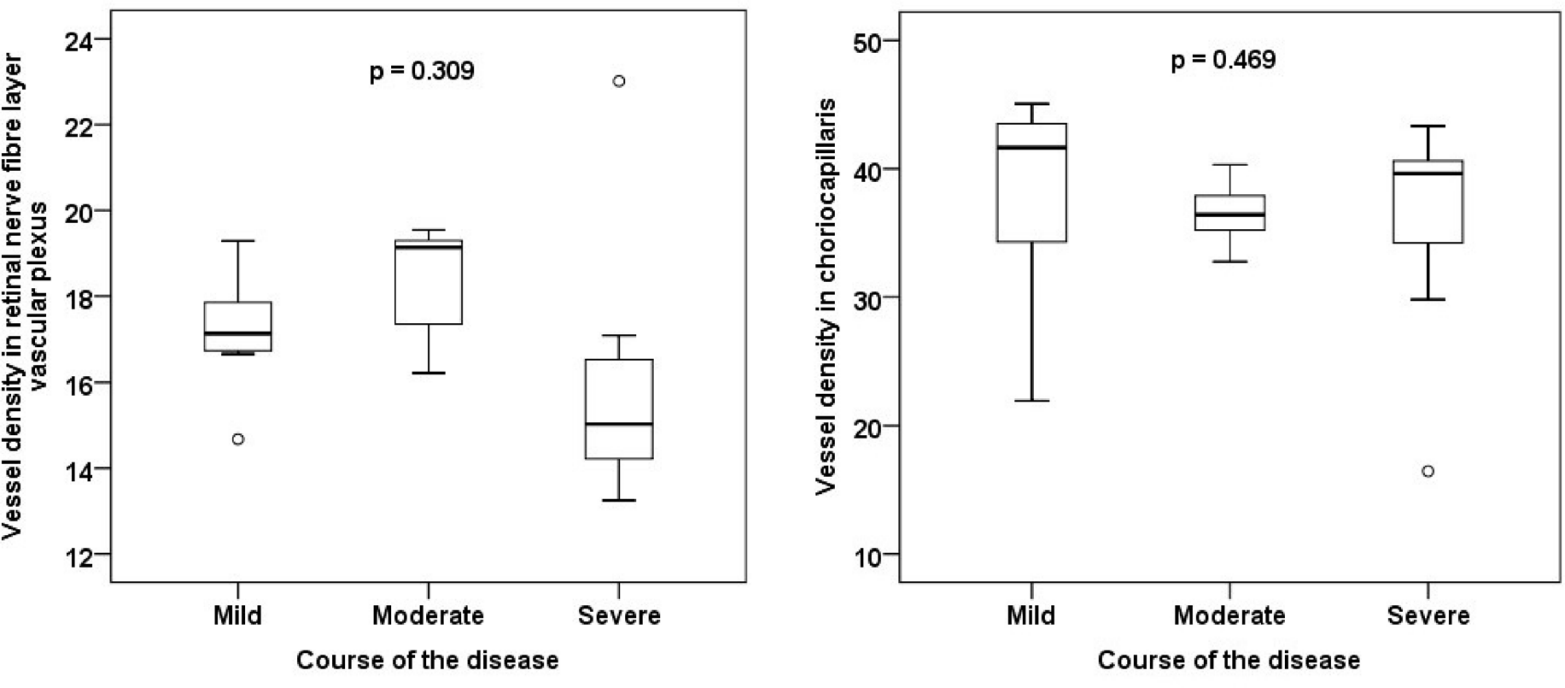
Comparison of vessel density in the retinal nerve fibre layer vascular plexus and choriocapillaris.

No statistically significant difference in the FAZ size, circumference, circularity and roundness or in the VD in the individual capillary plexuses were found between the groups (Figs. 4, 5, 6 and 7). No correlation between AV difference and FAZ or between AV difference and VD in individual groups was detected.

## Discussion

While RO is the focus of mainly vascular or neurological diseases such as DM, multiple sclerosis or Alzheimer’s disease^37–40^, respiratory diseases are often overlooked. COVID-19 is primarily a pulmonary disease leading to endotheliitis, thus inducing a prothrombotic state with terminal arteriole occlusion and ischaemia. This situation calls for the investigation of retinal oxygen saturation, especially in individuals after recovery. The absence of difference in the diameter of arteries and veins and in oxygen saturation between groups based on COVID-19 severity is in accordance with the recent study of Austrian authors^24^. Researchers monitored retinal vessel diameters and retinal oxygen saturation using a dynamic vessel analyser, blood flow through the eye using laser speckle flowgraphy and VD using Heidelberg Spectralis OCT (Heidelberg Engineering, Heidelberg, Germany). The study is based on the comparison of a group of 29 subjects with moderate to severe COVID-19, recovery time of 2–23 weeks, with 11 healthy controls. No significant differences in the diameter of the arteries and veins and in arterial or venous oxygen saturation were detected, but the AV difference was statistically significantly lower in post-COVID-19 patients than in healthy individuals. VD of large vessels was significantly lower in post-COVID-19 subjects than healthy controls. On the other hand, no difference was found in VD in SVP, ICP or DCP. The authors did not measure FAZ and their cohort did not include patients after ECMO.

To the best of our knowledge, ocular symptoms in the retina of adults after ECMO was solely analysed by Blegen^41^. They performed a retrospective analysis of biomicroscopic fundus findings in 20 patients after acute respiratory distress syndrome, who were followed during the nearly seven-year period of the study. They reported intraocular haemorrhage, Purtscher-like retinopathy and infectious chorioretinitis. Microvascular alterations—FAZ or VD—were not investigated. However, their study focused on acute retinal findings, not on long-term changes as ours.

A statistically significant positive correlation between the AV difference and IRL thickness may be explained by the different oxygen demand in the different retinal layers. Authors^42^ found that there are three layers in the rat retina with the greatest oxygen consumption—the inner segments of the photoreceptors, the outer plexiform layer (OPL), and the deeper region of the inner plexiform layer (IPL). This could be related to our results; the greater the oxygen consumption in the retina, i.e., the arterio-venous difference, the greater the thickness of the IRL, which includes both the OPL and the deeper region of the IPL.

Macular VD depends on many factors, i.a. age^43,44^ or obesity^45^. Some authors reported that cigarette smoking was associated with lower subfoveolar retinal thickness, a larger FAZ area^46^ and lower VD^47,48^. Other studies failed to find this association^49,50^. Wang evaluated the VD effect on visual quality in young myopes^51^. Axial length (AL) was negatively correlated with macular VD in some of the SCP regions and with FAZ size. Our study did not consider AL. However, we excluded patients with a significant refractive error (a refractive error greater than ± 6.0 spherical dioptres or ± 3.0 cylindric dioptres) and the value range of refractive errors in our study was from -2.375 to + 3.375 dioptres of spherical equivalent, therefore our results should not be affected by this factor.

Wang^52^ examined open-angle glaucoma patients to find out whether VD-associated changes develop throughout the day and to compare these patients with healthy controls. Macular VD in SCP and DCP varied significantly more during the day in POAG patients than in healthy controls, pointing to impaired vascular autoregulation in POAG. In our cohort, VD measurements were performed in patients independently of the time of day, which should not be a misleading factor based on the results of the previous study.

Several studies analysed VD and FAZ in individuals after COVID-19. Hazar examined a group of 50 cases one month after COVID-19 pneumonia against a control group of 55 healthy individuals^19^. They found reduced VD in certain capillary plexuses in some of the macular quadrants in patients compared to controls. However, no statistically significant differences between groups were detected in VD in the entire OCT-A scan or in FAZ size, which is also consistent with our results.

González-Zamora compared OCT and OCT-A parameters in patients with bilateral COVID-19 pneumonia against healthy controls^18^. The results revealed changes in VD and FAZ in SVC, in the DVC and thinner GCL in the fovea, as well as thicker RNFL at the ON disc in patients after pneumonia. No statistically significant differences in these parameters were detected in our group, possibly due to the lower average patient age, as VD decreases and FAZ increases with higher age^43,44^. A borderline difference in the RNFL parameter at the ON disc was found between group 1 versus 2 (*p* = 0.078, Kruskal–Wallis test and Bonferroni correction) and 1 versus 3 (*p* = 0.064). Spanish authors failed to demonstrate differences in CRT and RNFL in the fovea, in FAZ size in DVC, or in VD CC, in accordance with our results. Parameters were measured using the DRI OCT Triton SS-OCT Angio (TopCon Medical Systems, Inc. Oakland, NJ, USA), enabling macular division into sectors according to the ETDRS grid, with VD subanalysis in these fields. Contrary to our study, they did not compare VD in the entire OCT scan.

The design of our study is most similar to Zapata^22^. The authors only compared FAZ and VD size in SVC in 96 individuals divided into 4 groups—mild, moderate and severe course of COVID-19, and healthy controls. Patients with moderate and severe disease course were found to have a significantly reduced VD in two macular sectors in SVC compared to mild disease course and controls. No statistically significant alterations in other sectors were found and FAZ size changes were not confirmed between the groups either. The study was conducted within 3 months of COVID-19 diagnosis, i.e., much earlier than our study, therefore, this factor may have influenced the results.

Similar results were reported using the same OCT device (Heidelberg HRA + OCT Spectralis System, Heidelberg Engineering, Heidelberg, Germany) by American authors^21^. They compared healthy controls with patients after outpatient COVID-19 management and with patients who required hospitalisation at an intensive care unit with respiratory support. VD in ICP and DCP were statistically significantly lower in patients after severe COVID-19 compared to patients with mild course and healthy controls. Nonetheless, no difference in VD in NFLVP and SVP or in retinal thickness or FAZ size was found between groups. Compared to the presented study, the number of subjects was similar, but patients with arterial hypertension (HT) history were excluded.

OCT-A results can be affected by multiple factors. Leaving aside pre-existing systemic diseases, one of the potential influencing factors is oxygen treatment. Previous studies postulated that retinal VD increases in response to hypoxemia and decreases in response of hyperoxemia in the acute phase of COVID-19 pneumonia. However, direct effects of lack or excess of oxygen last only a few hours or days^53,54^. So, it is possible that as the interval from the onset of the acute disease increases, the inflammatory changes and the effects of hyperoxia/hypoxia diminish so that long-term damage does not occur. In addition, the retinal circulation has no autonomic innervation and its regulatory mechanisms are not under neurogenic control, but under the control of local mediators released by the endothelium of blood vessels or retinal cells^55^. On this basis, we conclude that the intrinsic autoregulatory mechanisms of the retina are capable of sufficient oxygenation to prevent long-term ischemic damage and the development of chronic retinal changes in almost all patients after COVID-19, regardless of the severity of the disease^55,56^.

The presented study is, to the best of our knowledge, the first to investigate RO in post-COVID-19 patients using Oxymap T1 more than 1.5 years (median 22 months) after COVID-19 recovery. In addition, patients after MV in the most severe phase of the disease or ECMO were included as well. There was a total of 9 people managed with ECMO in our cohort, which is a reasonably high number given the high mortality of these critically ill patients. The most important finding of our study is the fact that patients after full recovery from severe COVID-19 requiring MV or ECMO do not show any significant decrease in oxygen saturation of large vessels, nor any significant structural changes in the macula or retinal microcirculation 1.5 years after the episode. Our data show that even COVID-19 with critical course does not lead to chronic microvascular changes in the retina.

This study is the first to combine RO with VD and FAZ measurement using OCT-A in patients after COVID-19. OCT-A is a modern non-invasive method enabling an automatic segmentation of retinal capillary plexuses in the macular area. This enables the monitoring of microvascular changes, such as microaneurysms, choroidal neovascularisation or changes in VD or FAZ size. With OCT, they provide an ideal way of retinal structure imaging. In combination with RO, it allows for a more detailed evaluation of retinal oxygen metabolism. This parameter could serve as a potential biomarker of organ damage, especially the central nervous system, because the retina and brain have similar blood flow—both lack autonomic innervation of blood vessels and both have their own control mechanisms to maintain constant blood flow. Retinal microvascular changes reflect the cerebral microvasculature in aging and diseases like cognitive impairment or HT, so we assume that these similarities could also be present in post-COVID-19 cases^57^. Given that we did not demonstrate an effect of COVID-19 on retinal microvasculature in our study, this would be positive news for individuals experiencing post-COVID-19 syndrome.

A further advantage of this study is the relatively low age of patients, the small number of their comorbidities and a balanced number of patients with HT, DM, dyslipidaemia and smoking history between the groups. Therefore, our results should not be significantly influenced by these factors.

A major limitation of the presented study is the relatively small cohort size, especially for OCT-A analysis. There are relatively few individuals who recovered from a severe COVID-19 course after MV or ECMO. Larger numbers of patients in other groups would preclude comparisons. Only one eye of each patient was analysed to avoid bias. Furthermore, patients with a DM history were excluded from OCT-A analysis, as even DM patients without diabetic retinopathy signs have a larger FAZ and lower VD compared to healthy controls^58–60^. Another inherent fact is the absence of recommended protocol for the procedure and analysis of RO in macular microcirculation. The correlation of OCT and OCT-A parameters with RO parameters directly in the central retinal area could be of interest, enabling a more detailed evaluation of oxygen metabolism.

It would be interesting to focus on changes in the active phase of the disease and their follow-up over time, which would help to clarify the exact evolution of any pathologies on RO and/or OCT-A. However, this was not possible due to the nature of the disease, its high infectivity, epidemiological measures and the poor general condition of the patients during ongoing COVID-19. In the future, a longitudinal study starting in the active phase of infection would certainly be needed, of course, if anti-epidemic measures are followed.

## Conclusion

The presented study confirmed the absence of differences in RO, OCT and OCT-A parameters between groups depending on COVID-19 severity. No changes in retinal vessel oxygenation, VD and FAZ parameters were detected even in critically ill patients after ECMO or MV, which could be considered a favourable outcome. Our findings suggest no persistent retinal microvascular changes.

## Data Availability

The datasets generated during and/or analysed during the current study are available from the corresponding author on reasonable request.
